# Design and expert validation of the OEPR learning model for sea turtle conservation educational tourism in Pacitan, Indonesia

**DOI:** 10.12688/f1000research.191070.1

**Published:** 2026-10-06

**Authors:** Wahyu Prihanta, Yuni Pantiwati, Abdulkadir Rahardjanto, Poncojari Wahyono

**Affiliations:** 1 Doctoral Program in Education, Universitas Muhammadiyah Malang, Malang, East Java, Indonesia; 2 Department of Biology Education, Universitas Muhammadiyah Malang, Malang, East Java, Indonesia

**Keywords:** sea turtle conservation; educational tourism; experiential learning; environmental education; model development; expert validation

## Abstract

**Background:**

Sea turtle conservation sites can function as authentic learning environments, but educational visits frequently remain informal, knowledge-centred, and disconnected from sustained participation. This study developed and established the initial content validity of an experiential learning model for conservation-based educational tourism at Taman Beach, Pacitan, Indonesia.

**Methods:**

An educational research-and-development study combined a preliminary needs assessment, document and literature analysis, field observation, stakeholder interviews, model design, and expert validation. Qualitative evidence was organised into six design needs. The resulting Orientation–Exploration–Practice–Reflection (OEPR) model was reviewed independently by experts in education, environmental education, and sea turtle conservation using five-point instruments covering syntax, social system, reaction principles, support system, model effects, model handbook, and facilitator guide. Scores were summarised as percentages, while written comments were analysed thematically and converted into revision decisions.

**Results:**

The needs assessment identified fragmented visit sequences, mainly lecture-based interpretation, limited media, weak community coordination, no structured learner assessment, and no systematic follow-up. Eleven years of monitoring records contained 14,441 eggs and 11,892 hatchlings; annual hatching proportions varied substantially, reinforcing the need to connect education with authentic monitoring data. Expert validity was 98.33% for education, 96.94% for environmental education, and 98.24% for conservation. Reviewers retained the four-stage syntax but strengthened sense of place, inquiry, social–ecological analysis, authentic practice, standard operating procedures, safety, scaffolding, reflection, and action planning.

**Conclusions:**

OEPR offers a coherent, place-based structure for transforming a conservation visit into an experiential and reflective learning cycle. The evidence supports content validity and readiness for feasibility and pilot testing, but not yet effectiveness. Subsequent studies should evaluate implementation fidelity, learner outcomes, and ecological safeguards with larger participant samples.

## Introduction

Sea turtles are ecologically important, highly mobile marine vertebrates whose conservation depends on the integrity of both oceanic and nesting-beach habitats. Although global conservation programmes have generated important recoveries, populations remain exposed to by-catch, coastal development, pollution, climate-driven changes in nesting conditions, disease, and direct exploitation ( Mazaris et al., 2017; Lovich et al., 2018; Barrios-Garrido et al., 2019; Mashkour et al., 2020; Lockley & Eizaguirre, 2021; Carpio et al., 2022; Costa et al., 2023; Dias et al., 2024). These interacting pressures mean that technical protection of nests and hatchlings is necessary but insufficient. Conservation also requires informed public behaviour, community participation, institutional continuity, and locally credible learning opportunities.

Conservation sites and nature-based tourism destinations can provide such opportunities because visitors encounter organisms, habitats, practitioners, and management dilemmas directly ( Hudha & Husamah, 2019; Husamah et al., 2024; Nurwidodo et al., 2026). Educational tourism and field-based learning may connect abstract concepts with lived experience, strengthen affective engagement, and support global and sustainability learning ( Arcodia et al., 2020; Chen et al., 2022; Husamah et al., 2025a; McGladdery & Lubbe, 2017). Sea turtle tourism is especially promising because nesting, hatchery management, hatchling care, beach protection, and release events are highly visible. However, wildlife encounters do not automatically produce conservation learning. Outcomes depend on interpretation quality, programme structure, ethical safeguards, facilitation, and opportunities to translate interest into informed action ( Busaidi et al., 2018; Nurhayati et al., 2022; Nurwidodo et al., 2023; Mascovich et al., 2023; Ramdiah et al., 2018). Poorly managed activities may instead disturb animals, simplify complex conservation problems, or reduce participation to symbolic events.

Environmental education research increasingly emphasises action competence rather than knowledge acquisition alone. Knowledge, attitudes, responsibility, social expectations, perceived agency, and opportunities for participation interact in shaping pro-environmental behaviour ( Lee & Jan, 2018; Nguyen et al., 2019; Chen & Liu, 2020; Begum et al., 2021; O’Halloran & Silver, 2022). Place-based and experiential pedagogies are particularly relevant because learners investigate real social–ecological conditions, act within a community setting, and reflect on the consequences of their decisions ( Ajaps & Mbah, 2022; Morris, 2020; Rahardjanto et al., 2025). Citizen-science and participatory approaches further show that authentic data collection and collaboration can support learning and conservation engagement when roles, feedback, and expected outcomes are made explicit ( Lewandowski & Oberhauser, 2016; Mitchell et al., 2017; Phillips et al., 2018).

At Taman Beach in Pacitan, East Java, sea turtle protection has developed alongside visits by schools and the public. The available records document patrols, egg relocation to a semi-natural hatchery, hatchling care, release activities, beach cleaning, and informal explanations by conservation personnel. Yet the source documents also indicate that visits have not followed a consistent pedagogical sequence; interpretation has relied mainly on oral explanation; community members have not been systematically prepared as educators; and learning has rarely been assessed or followed up. This gap is both practical and conceptual. Existing tourism, experiential-learning, and community-conservation approaches each provide useful principles, but they do not by themselves specify an operational learning syntax for this local sea turtle conservation setting.

Accordingly, this study asked: (1) What empirical needs should guide a conservation-based educational tourism model at Taman Beach? (2) How can those needs be transformed into an operational learning model? and (3) To what extent is the initial model judged valid by experts in education, environmental education, and sea turtle conservation? The study aimed to develop the OEPR model—Orientation, Exploration, Practice, and Reflection—and establish its initial content validity. Its contribution is a compact, field-operable learning cycle that integrates sense of place, inquiry, authentic conservation practice, community partnership, safety, and reflective action planning.

## Methods

### Study design

The study used an educational research-and-development design focused on the analysis, design, and expert-validation stages. It did not test causal effectiveness. Development proceeded through six linked activities: (1) synthesis of existing research and local documents; (2) field observation of conservation and educational-tourism practices; (3) in-depth stakeholder interviews; (4) identification of gaps and core needs; (5) design of the model and supporting products; and (6) multidisciplinary expert validation followed by revision. This sequence was used to maintain traceability from empirical evidence to design decisions.

### Setting and source materials

The development context was the sea turtle conservation area at Taman Beach, Pacitan Regency, East Java, Indonesia. The evidence corpus comprised: field-observation notes; an in-depth interview matrix organised around six needs-assessment domains; conservation monitoring records; photographic documentation of the entrance, office, hatchery, hatchling pools, quarantine pool, visitor gazebos, educational visits, releases, and observations; literature-analysis notes; and draft model-development documents. Monitoring data covered 2013–2022 and 2024; no 2023 value was present in the supplied record.

### Needs assessment and qualitative analysis

Observation examined conservation principles, educational interpretation, community participation, potential curriculum content, local context and innovation, and current evaluation practice. Interviews explored ongoing conservation work, challenges, educational activities, community roles, suitable visitor activities, sequencing, local knowledge, innovation, and evaluation. Evidence was condensed, compared across sources, and organised into themes. A design need was retained when it was supported by at least two forms of evidence or when it represented a safety, ethics, or conservation requirement. The resulting needs were mapped to model objectives, learning phases, actor roles, support requirements, and evaluation targets.

### Model construction

The model combined experiential learning, constructivist and contextual learning, place-based environmental education, social learning, and participatory conservation. Four phases were chosen to balance pedagogical completeness with field feasibility: Orientation establishes readiness, purpose, prior knowledge, place attachment, and rules; Exploration organises inquiry into ecological and social conditions; Practice provides authentic, supervised conservation action; and Reflection supports meaning-making, feedback, commitment, and an action plan. The draft product also specified a social system, facilitator reaction principles, support system, intended instructional and nurturant effects, a model handbook, and a facilitator guide.table.

### Expert validation

Three reviewers were selected purposively to provide complementary expertise: one in education, one in environmental education, and one in sea turtle conservation practice. Each independently assessed seven components—OEPR syntax, social system, reaction principles, support system, model effects, model handbook, and facilitator guide—on five-point scales. The education instrument contained 96 items, the environmental-education instrument 98 items, and the conservation instrument 91 items. Component and total percentages were calculated as obtained score divided by maximum possible score multiplied by 100. Written comments were compared thematically and assigned one of four decisions: retain, strengthen, enrich without changing the core construct, or do not adopt as a construct change.

### Data analysis

Qualitative evidence was analysed through data condensation, thematic display, cross-source comparison, and conclusion drawing. Quantitative validation used descriptive totals, mean item scores, and percentages. Conservation records were summarised as annual counts and hatching proportions. Because the study used three domain experts rather than a participant sample, no inferential tests or inter-rater reliability coefficients were calculated. The unit of inference was initial content and design validity, not learning effectiveness.

## Results

### Conservation and educational-tourism context

Existing conservation work included seasonal beach patrols, recovery and relocation of eggs to a protected semi-natural hatchery, marking of relocated clutches, transfer of hatchlings to holding pools, seawater replacement and feeding while temporarily held, and release to the sea. Community members sometimes reported or handed over eggs, participated in beach cleaning, helped protect nests, and assisted during release events. The principal reported constraints were limited personnel, illegal egg collection by some individuals, plastic pollution, habitat disturbance, limited facilities, and weak organisational support.

As shown in Table 1, the supplied records contained 14,441 eggs and 11,892 hatchlings across 11 reported years, equivalent to an aggregate proportion of 82.3%. Annual proportions ranged from 15.1% in 2015 to 96.2% in 2024. These values are descriptive operational records rather than controlled biological estimates: clutch-level covariates, relocation intervals, incubation conditions, losses, and denominator definitions were not available. The marked annual variability nevertheless demonstrates the educational value of authentic monitoring data and the need to teach learners how to interpret conservation indicators cautiously.

**Table 1.** Annual numbers of sea turtle eggs and hatchlings recorded at Taman Beach, with calculated hatching proportions.

Year	Eggs	Hatchlings	Hatching proportion (%)
2013	187	151	80.7
2014	166	149	89.8
2015	776	117	15.1
2016	224	205	91.5
2017	311	298	95.8
2018	1,205	986	81.8
2019	1,317	871	66.1
2020	1,302	1,095	84.1
2021	2,757	2,172	78.8
2022	917	769	83.9
2024	5,279	5,079	96.2
Total	14,441	11,892	82.3

### Six core needs

Triangulation produced six design needs ( Table 2). Educational visits already contained potentially powerful experiences—hatchery observation, hatchling viewing, beach cleaning, and release—but the sequence was inconsistent and objectives were concentrated on factual knowledge. Visitors usually received a lecture while walking around the facility. Structured welcoming activities, inquiry tasks, discussion, assessment, reflection, and follow-up were absent or inconsistent. Media were limited, guides were not coordinated through a shared script, and community–visitor interaction mostly concerned commerce rather than conservation learning.

**Table 2.** Empirical needs and corresponding design responses incorporated into the OEPR learning model.

Core need	Empirical basis	Design response
Conservation integrity	Protection of nesting turtles, eggs, hatchlings and habitat; compliance with SOPs; safe timing and handling; accurate monitoring data.	Embed non-negotiable SOP and safety checkpoints in every phase.
Structured environmental learning	Interpretation was mainly oral, incidental and knowledge-focused; visual/digital media and level-appropriate modules were limited.	Use explicit objectives, inquiry prompts, media, worksheets and differentiated facilitation.
Meaningful community participation	Community members contributed to patrols, egg reporting, cleaning and releases but were not consistently prepared as educators.	Position community members and conservation staff as learning partners with defined roles.
Authentic curriculum sequence	Hatchery observation, habitat exploration, release and beach care were suitable but not organised into a learning cycle.	Sequence readiness, investigation, supervised action and reflection.
Place-responsive innovation	Local stories, coastal ecology and educational-tourism ideas were present but not integrated pedagogically.	Use sense of place and locally appropriate content without commodifying wildlife.
Evaluation and continuity	No structured learner indicators, documentation system or post-visit action plan was used.	Assess knowledge, attitudes and participation; document learning and establish follow-up actions.

### The initial OEPR model

The evidence-to-design process resulted in a four-stage cycle ( Figure 1). Orientation activates prior knowledge and motivation, introduces the conservation site and its social–ecological significance, clarifies ethical and safety rules, and distributes participant roles. Exploration guides learners to observe habitats and facilities, ask questions, examine monitoring evidence, and analyse ecological and community dimensions. Practice involves supervised, SOP-compliant activities appropriate to season and participant capacity, such as beach cleaning, habitat observation, data recording, or carefully managed hatchling-release support. Reflection structures discussion, checks understanding, links experience to everyday decisions, and concludes with feasible individual or collective action commitments.

**Figure 1.**
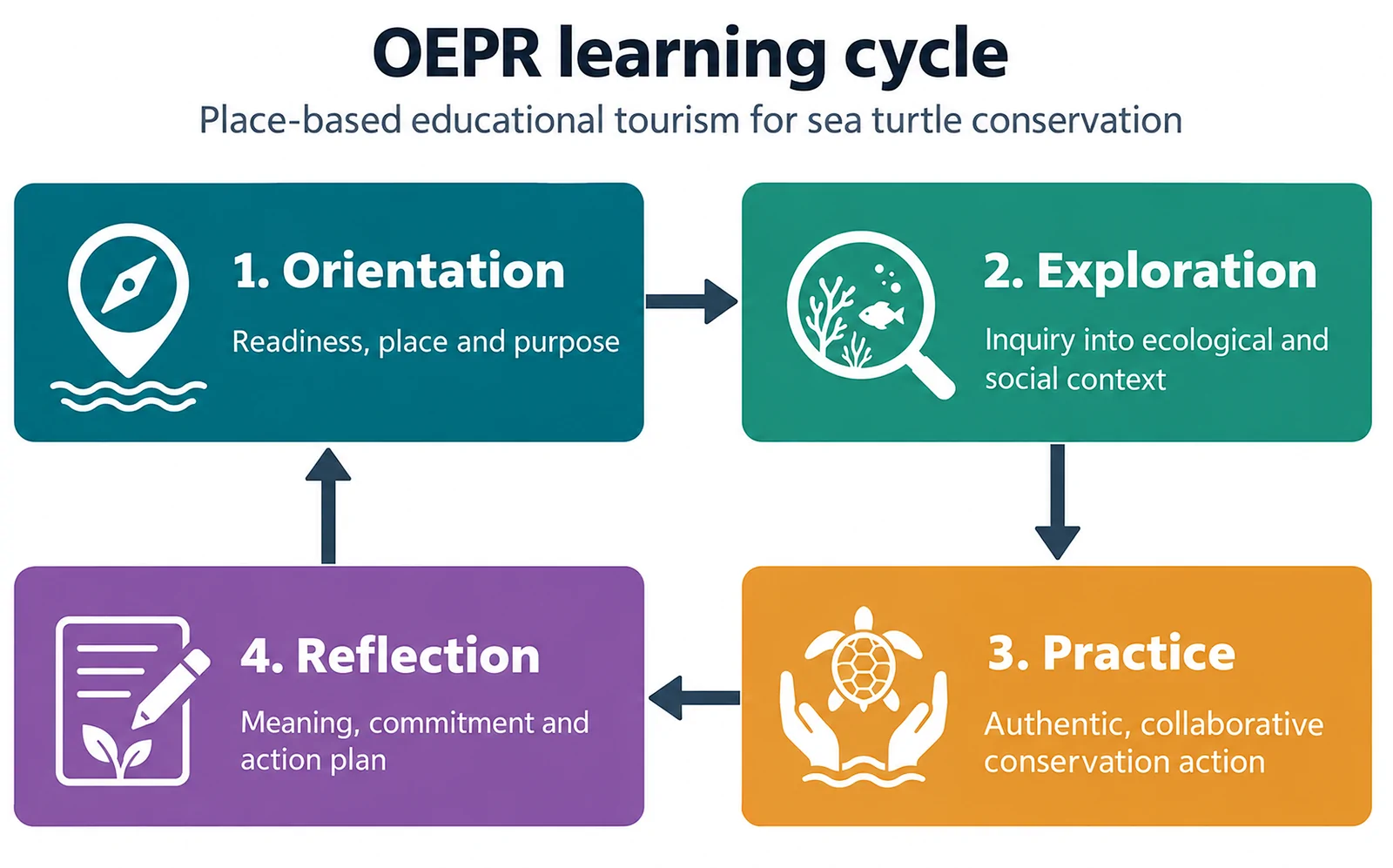
Post-validation Orientation–Exploration–Practice–Reflection (OEPR) learning cycle for sea turtle conservation educational tourism. Arrows indicate the iterative sequence from learner preparation and place orientation, through inquiry and supervised conservation practice, to structured reflection and action planning.

### Expert-validation outcomes

The overall expert-validation results are presented in Table 3, and the component-level validity percentages are detailed in Table 4. All three reviews placed the draft in the highest validity category used in the development documents. Component scores were consistently high. The education expert assigned 94.55–100.00% across components; the environmental-education expert assigned 96.00–97.50%; and the conservation expert assigned 97.22–100.00%. These results indicate strong agreement that the model components were relevant and coherent, while the written comments identified important operational refinements.

**Table 3.** Overall expert-validation scores for the OEPR learning model by area of expertise.

Expert perspective	Items	Obtained score	Maximum score	Mean item score	Validity (%)
Education	96	472	480	4.92	98.33
Environmental education	98	475	490	4.85	96.94
Sea turtle conservation	91	447	455	4.91	98.24

**Table 4.** Component-level validity percentages assigned by the education, environmental-education, and sea turtle conservation experts.

Component	Education	Environmental education	Conservation
OEPR syntax	98.92	97.22	97.22
Social system	100.00	97.14	97.78
Reaction principles	98.00	97.50	97.78
Support system	100.00	96.00	100.00
Model effects	100.00	96.67	97.78
Model handbook	96.36	97.33	100.00
Facilitator guide	94.55	96.25	100.00

### Revision decisions and post-validation model

The four-stage order was retained. Orientation was strengthened by adding readiness, sense of place, motivation, and a clear bridge to Exploration. Exploration was specified as inquiry into both ecological and social–ecological conditions. Practice was reframed as authentic and collaborative conservation action bounded by SOPs, animal welfare, participant safety, and site capacity. Reflection was strengthened through structured prompts, formative feedback, commitment, and an action plan. The social system clarified the facilitator’s responsibility and positioned conservation staff and community members as partners and sources of knowledge. Reaction principles added adaptive scaffolding, corrective feedback for unsafe or non-compliant conduct, and support for field documentation. The support system was revised around sufficiency, contextual fit, integration, flexibility, safety, periodic evaluation, and updating.

Three primary instructional effects were retained: conservation knowledge, conservation attitudes, and conservation participation. Practical skills were treated as enabling capabilities rather than a fourth primary outcome. Environmental care, sustainability awareness, and conservation commitment remained nurturant effects. Digitalisation, inquiry skill, stewardship, leadership, and broader transfer to daily life were incorporated as adaptable enrichments rather than mandatory new constructs. Proposed changes that would shift the study into destination governance or institutional development were not adopted because they exceeded the boundaries of a learning model.

## Discussion

The study shows that the main weakness at the case site was not an absence of conservation activity, but a gap between conservation activity and deliberate learning design. Hatchery visits, beach care, monitoring, and release events already created authentic experiences. Yet authenticity alone does not guarantee learning. Without explicit questions, evidence use, skilled facilitation, feedback, and reflection, a visit may remain memorable but educationally shallow. This distinction is consistent with educational-tourism research showing that field experience becomes learning when activity and meaning-making are intentionally connected ( McGladdery & Lubbe, 2017; Arcodia et al., 2020; Morris, 2020).

OEPR responds by converting a collection of activities into a short but theoretically defensible cycle. Orientation prevents the common mistake of beginning immediately with animal encounters; it establishes purpose, ethical limits, and place meaning. Exploration adds inquiry and ensures that learners encounter not only charismatic hatchlings but also habitat, pollution, monitoring, human livelihoods, and management constraints. Practice makes participation real while protecting animals through supervision and SOPs. Reflection addresses the well-documented gap between positive environmental attitudes or intentions and sustained behaviour ( Nguyen et al., 2019). In this respect, OEPR aligns with action-competence approaches that treat learners as capable participants rather than passive recipients of conservation messages ( Chen & Liu, 2020).

The model also treats community involvement as pedagogical infrastructure. Community-based conservation can gain legitimacy and continuity when local actors have meaningful roles, but participation is weakened when residents are invited only to implement externally defined activities ( Murray & Agyare, 2018; Arnold et al., 2022; Petriello et al., 2025). At Taman Beach, residents already possessed experience in nest reporting, beach conditions, past exploitation, and visitor interaction. Defining community members and conservation personnel as learning partners can make these forms of knowledge visible, while common facilitator guidance can reduce inconsistency and misinformation. This approach should not romanticise local knowledge or obscure power and livelihood tensions; rather, it creates a structured opportunity to examine them.

The monitoring record adds an important data-literacy dimension. Annual hatching proportions varied widely and 2023 was missing. Such records can support authentic questions about incubation conditions, relocation, beach disturbance, and data quality, but they should not be used to claim trends without clutch-level metadata and consistent definitions. Integrating real data into Exploration and Reflection can help learners understand that conservation success is measured, uncertain, and affected by both ecological and operational factors. Comparable long-term monitoring research demonstrates the value of consistent nest records while also showing why interpretation depends on methods and environmental covariates ( Bomfim et al., 2021; Patrício et al., 2019; Husamah et al., 2025b).

The high expert scores support content coherence across three distinct perspectives, and the comments improved boundary conditions that a purely pedagogical review might overlook. Conservation review strengthened SOPs, safety, and animal welfare; environmental-education review strengthened place, inquiry, social–ecological relations, and adaptability; and education review strengthened operational clarity, scaffolding, and usability. This complementarity is more informative than the percentages alone. However, high ratings should not be interpreted as evidence that OEPR improves learner outcomes. Expert validation is an early design-quality test, and favourable ratings can be influenced by the small panel, instrument length, and shared expectations about the product.

Before wider use, the model should be tested in staged cycles. A feasibility study should examine timing, guide preparation, seasonal availability of activities, safety compliance, participant comprehension, and burden on conservation staff. A pilot should then assess implementation fidelity and short-term changes in knowledge, attitudes, and intended or observed participation, using validated instruments and qualitative feedback. A comparative or controlled evaluation would be appropriate only after delivery is stable. Ecological safeguards should remain explicit: educational demand must never determine wildlife handling, holding time, release timing, lighting, crowd size, or access to sensitive nesting areas.

A strength of this study is the traceable connection between local evidence, theoretical principles, model components, and expert revisions. The expert panel covered pedagogy, environmental education, and conservation practice, reducing the likelihood that usability would be prioritised over ecological integrity. The study also incorporated operational monitoring and photographic documentation rather than relying only on perceptions. Limitations include a single-site context, only three expert reviewers, incomplete methodological metadata for conservation counts, no learner pilot or effectiveness data. Interview sampling characteristics and verbatim transcripts were not sufficiently detailed for claims about thematic saturation. Transfer to other sites therefore requires contextual adaptation and renewed conservation review.

## Conclusions

The OEPR model transforms sea turtle conservation educational tourism into a four-stage learning cycle: Orientation, Exploration, Practice, and Reflection. Its design arose from documented gaps in visit structure, media, guide coordination, community roles, learner evaluation, and follow-up. Three experts rated the initial model highly (96.94–98.33%) and retained its core syntax while strengthening place-based inquiry, authentic practice, SOP compliance, safety, scaffolding, reflection, and action planning. OEPR is therefore sufficiently coherent for feasibility and pilot testing. It should not yet be presented as effective; evidence of implementation fidelity, learner outcomes, sustained participation, and ecological safety remains necessary.

## Ethical considerations

The study was reviewed and approved by the Research Ethics Commission, Faculty of Education, Science, and Humanities, Universitas Muhammadiyah Malang, Indonesia (Komisi Etik Penelitian FPSH UMM; approval No. E.5.a/003/KEP-FPSH-UMM/VII/2026; 17 July 2026). Written informed consent was obtained from all adult participants before their participation in interviews, focus-group discussions, or expert validation. Participation was voluntary, participants could withdraw, and identifying information was excluded from the deposited materials. No minors participated. No research intervention was performed on sea turtles; the study documented existing conservation and educational practices.

## Data Availability

Figshare: *Underlying and extended data for ‘Design and expert validation of the OEPR learning model for sea turtle conservation educational tourism in Pacitan, Indonesia’.* https://doi.org/10.6084/m9.figshare.33868876 ( Prihanta, 2026). The Figshare record contains the following underlying data:
•
02_OEPR_Preliminary_Study_Data.xlsx (structured preliminary-study evidence derived from interviews, focus-group discussions, observation, documentation, and literature synthesis); •
03_OEPR_Monitoring_and_Observation_Data.xlsx (annual conservation monitoring records and structured field-observation data); •
04_OEPR_Expert_Validation_Data.xlsx (anonymised item-level ratings, component summaries, and reconciliation checks for the three expert validators); •
06_Anonymised_Qualitative_Source_Texts.docx (anonymised interview and focus-group source texts); •
08_Anonymised_Expert_Validation_Comments.docx (anonymised expert comments and recommendations). The same Figshare record contains:
• 00_README.txt; •
01_OEPR_Data_Dictionary_and_Manifest.xlsx; •
05_OEPR_Documentation_Register.xlsx; •
07_Figshare_Metadata.txt; and LICENSE.txt. The validation item wording and scoring structure are included in 04_OEPR_Expert_Validation_Data.xlsx. All publicly deposited files are available under the Creative Commons Attribution 4.0 International (CC BY 4.0) licence. Not applicable.
