# Supplementary material for: Design and expert validation of the OEPR learning model for sea turtle conservation educational tourism in Pacitan, Indonesia
Source: F1000Res. 2026 Oct 21;15:1674. Originally published 2026 Oct 6. [Version 2] doi: 10.12688/f1000research.191070.2 (PMC13658989; doi:10.12688/f1000research.191070.2)
Supplement: Peer review history

# Reviewer report: Reviewer response for version 1

**DOI:** 10.5256/f1000research.210886.r528475

**Published:** 2026-10-12

**Authors:** Supiana Dian Nurtjahyani

**Affiliations:** 1 Universitas PGRI Ronggolawe Tuban, Tuban, East Java, Indonesia

**General Assessment**

 The manuscript addresses an important intersection between conservation education and experiential learning by introducing the Orientation–Exploration–Practice–Reflection (OEPR) model. The study is relevant, logically organised, and supported by field-based evidence and multidisciplinary expert evaluation. Its particular strength lies in translating existing sea turtle conservation practices into a structured educational framework. The manuscript is generally suitable for indexing, although a few refinements would improve its clarity and practical relevance.

**Comments for the Authors**

 1. Introduction: The authors have established a convincing rationale for developing the OEPR model. It would be useful to include a brief explanation of how the proposed learning sequence responds to educational challenges specific to sea turtle conservation tourism, beyond those commonly addressed by experiential learning models.

 2. Methods: The development process is described systematically. A short clarification regarding how the six identified educational needs were prioritised during model construction would make the connection between the preliminary investigation and the final design more transparent.

 3. Results: The expert validation scores are consistently high and indicate favorable evaluations. The presentation could be improved by briefly explaining the practical significance of the differences across the seven validated components, rather than relying primarily on percentage scores.

 4. Discussion: The discussion effectively connects the findings with environmental education and conservation practices. The authors may consider adding a brief example illustrating how local conservation personnel or community members could facilitate one of the OEPR learning stages. This would help readers better understand the model's potential field application.

 5. Conclusion: The conclusion is consistent with the scope of the research. A concise statement identifying the immediate priorities for pilot implementation would further enhance its practical contribution.

 6. Editorial Aspects: Minor language and formatting adjustments are recommended to ensure consistency in terminology and presentation throughout the manuscript.

  The suggested improvements can be addressed through clarification and minor textual adjustments without changing the research design or requiring additional data collection.

Is the work clearly and accurately presented and does it cite the current literature?

Yes

If applicable, is the statistical analysis and its interpretation appropriate?

Yes

Are all the source data underlying the results available to ensure full reproducibility?

Yes

Is the study design appropriate and is the work technically sound?

Yes

Are the conclusions drawn adequately supported by the results?

Yes

Are sufficient details of methods and analysis provided to allow replication by others?

Yes

Reviewer Expertise:

Biology, Plant and Animal Conservation, Fungi, and Biology Education

I confirm that I have read this submission and believe that I have an appropriate level of expertise to confirm that it is of an acceptable scientific standard.

# Reviewer report: Reviewer response for version 1

**DOI:** 10.5256/f1000research.210886.r528469

**Published:** 2026-10-12

**Authors:** Endang Susantini

**Affiliations:** 1 Universitas Negeri Surabaya, Surabaya, Surabaya, Indonesia

The manuscript presents a relevant and well-structured study developing the Orientation–Exploration–Practice–Reflection (OEPR) learning model for sea turtle conservation educational tourism. The integration of experiential learning, community participation, and conservation practices is a valuable contribution. The research methodology is generally appropriate, and the findings are clearly presented. However, several minor improvements could further strengthen the manuscript's clarity, methodological transparency, and practical implications.

 Specific Comments

 1. Introduction: The introduction provides a sufficient theoretical background. However, the authors are encouraged to briefly clarify the distinctive contribution of the OEPR model compared with existing experiential and place-based learning approaches. This would make the study's novelty more explicit.

 2. Methods – Needs Assessment: The authors could provide brief additional information about the stakeholders involved in the interviews, particularly their roles and selection criteria. This clarification would improve methodological transparency without requiring further data collection.

 3. Methods – Expert Validation: The expert validation procedure is adequately described. Nevertheless, the authors are encouraged to briefly explain the rationale for selecting three experts and clarify the criteria used to interpret the validity percentages.

 4. Results: Tables 3 and 4 clearly present the expert validation outcomes. The authors could strengthen the interpretation by briefly highlighting which model components received relatively lower scores and explaining how the experts' suggestions informed their refinement.

 5. Discussion: The discussion provides a meaningful interpretation of the findings. It could be strengthened by adding a short explanation of how the OEPR model might be adapted to other conservation-based educational tourism settings while maintaining local ecological and cultural relevance.

 6. Conclusion: The conclusion appropriately acknowledges that the model has not yet undergone effectiveness testing. The authors could briefly specify the most important learning outcomes to be assessed in future pilot studies, particularly conservation knowledge, attitudes, and participation.

 7. Language and References: The manuscript is generally well written and supported by relevant literature. Minor proofreading is recommended to correct occasional typographical and formatting inconsistencies, particularly in the Methods section and reference list.

 The manuscript has a clear research focus, a relevant methodological approach, and potentially useful implications for conservation-based environmental education. The suggested revisions are relatively straightforward and can be addressed through clarification and refinement of the existing manuscript without additional experiments or major analyses. I recommend approval with minor revisions.

Is the work clearly and accurately presented and does it cite the current literature?

Yes

If applicable, is the statistical analysis and its interpretation appropriate?

Yes

Are all the source data underlying the results available to ensure full reproducibility?

Yes

Is the study design appropriate and is the work technically sound?

Yes

Are the conclusions drawn adequately supported by the results?

Yes

Are sufficient details of methods and analysis provided to allow replication by others?

Yes

Reviewer Expertise:

Biology education, biology, sustainability, ecology, and literacy

I confirm that I have read this submission and believe that I have an appropriate level of expertise to confirm that it is of an acceptable scientific standard.
